# Complete Genome Sequence of *Akkermansia* *glycaniphila* Strain Pyt^T^, a Mucin-Degrading Specialist of the Reticulated Python Gut

**DOI:** 10.1128/genomeA.01098-16

**Published:** 2017-01-05

**Authors:** Janneke P. Ouwerkerk, Jasper J. Koehorst, Peter J. Schaap, Jarmo Ritari, Lars Paulin, Clara Belzer, Willem M. de Vos

**Affiliations:** a Laboratory of Microbiology, Wageningen University, Wageningen, The Netherlands; b Systems and Synthetic Biology, Wageningen University, Wageningen, The Netherlands; c Institute of Biotechnology, University of Helsinki, Helsinki, Finland; d Department of Veterinary Biosciences, University of Helsinki, Helsinki, Finland; e RPU Immunobiology, Department of Bacteriology & Immunology, University of Helsinki, Helsinki, Finland

## Abstract

*Akkermansia glycaniphila* is a novel *Akkermansia* species that was isolated from the intestine of the reticulated python and shares the capacity to degrade mucin with the human strain *Akkermansia muciniphila* Muc^T^. Here, we report the complete genome sequence of strain Pyt^T^ of 3,074,121 bp. The genomic analysis reveals genes for mucin degradation and aerobic respiration.

## GENOME ANNOUNCEMENT

The ability to grow on mucin as a sole carbon and nitrogen source is a distinctive feature of the human isolate *Akkermansia muciniphila* Muc^T^ (1). *A. glycaniphila* strain Pyt^T^ is the second *Akkermansia* species described to be mucolytic in pure culture (2). Based on 16S rRNA gene sequences, the *Akkermansia* genus has recently been associated with the intestine or feces from a wide range of mammals and nonmammals, such as birds, fish, and reptiles, such as the Burmese python (3, 4). Here, we describe the complete genome sequence of *Akkermansia glycaniphila* strain Pyt^T^, an anaerobic mucin-degrading specialist isolated from the intestine of the reticulated python (*Malayopython reticulatus*).

Total DNA of strain Pyt^T^ was extracted using the MasterPure Gram-positive DNA purification kit (Epicentre). Single-molecule sequencing was performed using a PacBio RSII instrument at the Institute of Biotechnology (University of Helsinki, Finland). Assembly was performed with PacBio SMRT Analysis pipeline version 2.2 and the HGAP protocol (5). Default settings were used, except for the following adaptations: minimum subread length, 500; minimum polymerase read length quality, 0.80; minimum seed read length, 7,000; split target into chunks, 1; alignment candidate per chunk, 24; genome size, 3,000,000; target coverage, 30; overlapper error rate, 0.06; overlapper mini length, 40; and overlapper k-mer, 14. Annotation was carried out with an in-house pipeline consisting of Prodigal version 2.5 for prediction of protein-coding DNA sequences (CDSs) (6), InterProScan 5RC7 for protein annotation (7), tRNAscan-SE version 1.3.1 for prediction of tRNAs (8), and RNAmmer version 1.2 for prediction of rRNAs (9). Additional protein function predictions were derived via BLAST identifications against the UniRef50 (10) and Swiss-Prot (11) databases (download August 2013). Subsequently, the annotation was further enhanced by adding EC numbers via PRIAM version 2013-03-06 (12). Noncoding RNAs were identified using rfam_scan.pl version 1.04 on release 11.0 of the RFAM database (13).

The Pyt^T^ genome is composed of a single chromosome of 3,074,121 bp, with a G+C content of 57.6%. It contains 2,532 CDSs, all 21 tRNA genes, and three complete rRNA operons. For 72% (1,811) of the coding sequences, a function could be predicted. Analysis of the genome revealed the presence of numerous mucin-degrading enzymes, of which many are predicted to be secreted: 54 glycoside hydrolases, one glycosyl hydrolase, seven sialidases, and three sulfatases. The Pyt^T^ genome is predicted to encode a cytochrome *bd* ubiquinol oxidase, indicating the potential for aerobic respiration. This bacterium might use this in the oxic-anoxic interface of its probable habitat: the intestinal mucin layer, as recently has been experimentally verified for *A. muciniphila* Muc^T^ (14).

The availability of this genome will enhance the understanding of metabolic and physiological properties of members of the genus *Akkermansia* present in the intestinal tract of *Animalia* in general.

### Accession number(s).

The complete genome sequence of *Akkermansia glycaniphila* strain Pyt^T^ was deposited at EMBL-EBI under the accession number LT629973.
